# Post‐acute sequelae of COVID‐19 in cancer patients: Two cohorts in UK and Hong Kong

**DOI:** 10.1002/cam4.70134

**Published:** 2024-12-07

**Authors:** Eric Yuk Fai Wan, Shing Fung Lee, Jiayi Zhou, Vincent Ka Chun Yan, Francisco Tsz Tsun Lai, Celine Sze Ling Chui, Xue Li, Carlos King Ho Wong, Esther Wai Yin Chan, Ian Chi Kei Wong

**Affiliations:** ^1^ Centre for Safe Medication Practice and research, Department of Pharmacology and Pharmacy, Li Ka Shing Faculty of Medicine The University of Hong Kong Hong Kong China; ^2^ Laboratory of Data Discovery for Health (D24H) Hong Kong Science and Technology Park Hong Kong China; ^3^ Department of Family Medicine and Primary Care, School of Clinical Medicine, Li Ka Shing Faculty of Medicine The University of Hong Kong Hong Kong China; ^4^ Department of Radiation Oncology National University Cancer Institute, National University Hospital Singapore Singapore; ^5^ Department of Clinical Oncology, Tuen Mun Hospital New Territories West Cluster, Hospital Authority Tuen Mun Hong Kong; ^6^ School of Nursing, Li Ka Shing Faculty of Medicine The University of Hong Kong Hong Kong China; ^7^ School of Public Health, Li Ka Shing Faculty of Medicine The University of Hong Kong Hong Kong China; ^8^ Department of Medicine, School of Clinical Medicine, Li Ka Shing Faculty of Medicine The University of Hong Kong Hong Kong China; ^9^ Department of Pharmacy The University of Hong Kong‐Shenzhen Hospital Shenzhen China; ^10^ The University of Hong Kong Shenzhen Institute of Research and Innovation Shenzhen China; ^11^ Aston Pharmacy School Aston University Birmingham UK

**Keywords:** cancers, COVID‐19, CVDs, long‐term effect

## Abstract

**Background:**

Limited research exists on the risks and spectrum of complications in post‐acute phase of COVID‐19 in cancer patients. This study aimed to evaluate the post‐acute effects of COVID‐19 on different types of morbidities among cancer patients across two regions with different healthcare systems and dominant variants of COVID‐19.

**Materials and Methods:**

Cancer patients with COVID‐19 from the UK Biobank (UKB, *n* = 2230; March 16, 2020 to May 31, 2021; pre‐Omicron‐variants dominant) and electronic medical records in Hong Kong (HK cohort, *n* = 22,335; April 1, 2020 to October 31, 2022; Omicron‐variant dominant) were included. Each COVID‐19 case was randomly matched with up to 10 non‐COVID‐19 cancer patients based on age and sex. Follow‐up lasted until 31 August 2021 for UKB and 23 January 2023 for HK. Inverse probability treatment weighting balanced cohort characteristics. Cox regression evaluated the association of COVID‐19 with morbidities occurred 30 days post‐infection.

**Results:**

Cancer patients with COVID‐19 consistently showed significantly higher risk of major cardiovascular diseases (CVDs) [UKB: hazard ratio [HR] 1.8 (95% CI 1.3, 2.5); HK: HR 1.4 (95% CI 1.1, 1.8)], CVD death [UKB: HR 4.3 (95% CI 2.9, 6.2); HK: HR 1.7 (95% CI 1.3, 2.4)], and all‐cause mortality [UKB: HR 4.7 (95% CI 4.0, 5.5); HK: HR 1.6 (95% CI 1.5, 1.7)] in both cohorts despite the difference in dominant variants. Cancer patients at advanced ages or severely infected had higher all‐cause mortality risk. However, associations between COVID‐19 and CVDs became insignificant for fully vaccinated patients.

**Conclusion:**

COVID‐19 infection is associated with increased risks of CVDs and mortality in cancer patients. Fully vaccination may reduce the post‐acute effects of COVID‐19 on CVDs. This information may guide effective pre‐emptive measures to reduce COVID‐19‐related morbidities and mortality in cancer patients.

## 1 INTRODUCTION

The COVID‐19 pandemic has impacted the health and well‐being of millions of people worldwide.^1^ In addition to acute disease, some COVID‐19 patients continue to experience long‐lasting symptoms, referred to as post‐acute COVID‐19 syndrome or long COVID.^2^ While the definition of post‐acute COVID‐19 remains to be determined, it has been suggested to be clinical presentations that develop or persist beyond 3 or 4 weeks since the onset of acute symptoms of COVID‐19, after which replication‐competent SARS‐CoV‐2 can no more be isolated.^3^ The impact of COVID‐19 on post‐infection health is a concern when many countries have exited the acute phase of the pandemic.

The public health importance of understanding the impact of and post‐COVID sequelae on cancer survivors cannot be overstated. The majority of literature on the risk of post‐acute complications are conducted in general population.^4^, ^5^ Nevertheless, cancer patients and survivors are a vulnerable population who may have compromised immune systems, putting them at higher risk for severe COVID‐19 infection and death.^6^, ^7^ While most patients may recover within a short period after COVID‐19 infection, cancer patients may be particularly vulnerable to the post‐acute effects of COVID‐19 because of their weakened immune systems and other health issues. Moreover, the occurrence of post‐COVID sequelae may adversely affect the continuity of oncological treatment and survival outcome, independent of the cancer prognostic factors.^8^ Understanding post‐acute effects of COVID‐19 on cancer patients can help us to monitor the disease progression and develop better treatment strategies.

To date, limited studies have investigated the risks and spectrum of complications in post‐acute phase of COVID‐19 among cancer patients, and thus patients may not be able to receive adequate treatment timely.^9^ This gap in knowledge underscores the urgent need to prioritize research on the post‐acute effects of COVID‐19 on cancer patients so that we can develop targeted interventions to address post‐COVID sequelae in this population. Therefore, by identifying cancer patients with (exposed group) and without (unexposed group) COVID‐19 infection, we evaluated the occurrence and risk factors of different types of morbidities after acute phase of COVID‐19 among cancer patients in two regions with distinct genetic compositions, healthcare systems and degree of exposure to COVID‐19 variants (United Kingdom [UK] and Hong Kong [HK]), with the intent of minimizing potential biases. In HK, the majority of COVID‐19 patients were from the fifth wave of infection, which occurred after the importation of the highly transmissible Omicron variant since January 2022 and led to the largest number of infections and deaths compared to previous waves.^10^ In contrast, the infected participants from the UK Biobank (UKB) cohort were mostly from the first and second waves of infection prior to the occurrence of Omicron variant.^11^ We hypothesized that consistent results from both cohorts could increase the validity and generalizability of our findings.

## 2 MATERIALS AND METHODS

### 2.1 Study design and population

In this study, we recruited two cohorts of cancer patients from HK and UKB.

For the HK cohort, we extracted population‐based electronic medical records from the Hong Kong Hospital Authority (HA) and linked vaccination records from the Department of Health (DH). The Hong Kong HA, as the largest governmental organization, coordinates all public hospitals and primary care clinics. The HA health care services can be accessed by all HK residents (>7.4 million) covering around 80% of all routine hospital admissions.^12^ DH, the government agency in charge of healthcare policies and provision of healthcare services, manage vaccination records of all HK residents. Death records were obtained from the Hong Kong Deaths Registry. These population‐based databases have been used in previous studies on the long‐term effects of COVID‐19 infection and have been reported to be reliable.^13^

The UKB is an ongoing prospective study on over 500,000 volunteers aged 49–86 at recruitment.^14^ The dataset included various baseline measurements and health outcomes for more than 10‐year follow‐up. The details of the study design have been described elsewhere.^15^ COVID‐19 tests results, linked with UKB, were provided by Public Health England, Public Health Scotland and Secure Anonymized Information Linkage.^16^

**FIGURE 1:**
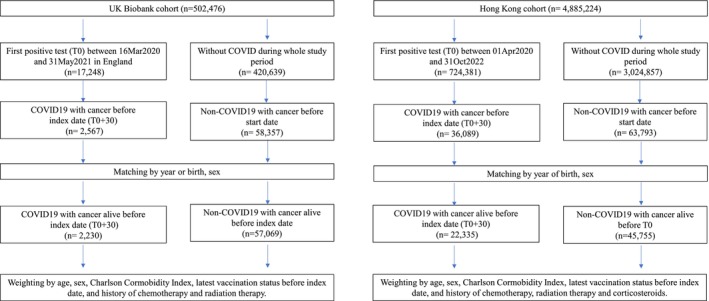
Flowchart of patients' selection.

A retrospective matched cohort study was conducted. The study period was from April 1, 2020 to January 23, 2023 for the HK cohort and from March 16, 2020 to August 31, 2021 for the UK cohort, which were the most up‐to‐date data available at the time of analysis. Patients with COVID‐19 infection (defined in the following text) were identified from April 1, 2020 to October 31, 2022 for the HK cohort and from March 16, 2020 to May 31, 2021 for the UK cohort. To study the post‐acute effects of COVID‐19 infection, only those who survived the acute‐phase of infection (30‐day period post‐diagnosis) were considered and identified as exposed group. The index date was therefore defined as 30‐day after COVID‐19 infection. For the HK cohort, patients who had no positive COVID‐19 test results (both Polymerase Chain Reaction (PCR) and Rapid Antigen Test) until January 23, 2023 were selected as unexposed group. For the UK cohort, the unexposed group were identified if patients were not tested positive by COVID‐19 PCR test, had no COVID‐19 diagnosis or any record of COVID‐19‐related deaths until October 18, 2021 (the date of latest valid record concerning COVID‐19 infections). We only included cancer patients aged 18 years or above who were diagnosed prior to the index date of COVID cases, as well as those who were diagnosed prior to the study's start date for the unexposed group (Figure 1). Each case in the exposed group was randomly matched with up to 10 people in the unexposed group by year of birth and sex, with identical index date assigned to the matched unexposed participants. All patients observed from the index date until their diagnosis of outcomes, the date of death or till January 23, 2023 for the HK cohort and August 31, 2021 for the UKB cohort, whichever was earliest.

### 2.2 Definition of cancer

Cancer was defined as any malignant neoplasm, except malignant nonmelanoma neoplasm of skin. The exclusion of malignant nonmelanoma neoplasm of skin is because it has a relatively high incidence but a low mortality, and is more common among people with fair skin tone; the definition was consistent with previous cancer studies.^17^, ^18^, ^19^ Cancer diagnoses were determined by the International Classification of Diseases, Ninth Revision, Clinical Modification (ICD‐9‐CM) codes of 140–171 and 174–209, and the International Classification of Diseases, Tenth Revision (ICD‐10) codes of C00.x‐C42.x, C45.x‐C96.x.

### 2.3 Definition of COVID‐19 infection

In the HK cohort, COVID‐19 infection was determined by PCR testing. In the UK cohort, infection was identified through a positive PCR test or a COVID‐19‐related hospital admission (codes: U07.1 and U07.2). COVID‐19 testing in the UKB consisted of two pillars: pillar 1 for healthcare professionals or those with clinical need, and pillar 2 for the broader population.

### 2.4 Outcome measures

Outcomes recorded after the index date included: (1) major cardiovascular disease (CVD) (heart failure, stroke, coronary heart disease (CHD)), (2) heart failure, (3) stroke, (4) CHD, (5) myocardial infarction (MI), (6) atrial fibrillation (and flutter), (7) deep vein thrombosis, (8) CVD death, (9) pleurisy or pleural effusion, (10) pulmonary embolism, (11) chronic pulmonary disease, (12) acute respiratory distress syndrome, (13) interstitial lung disease, (14) seizure, (15) Bell's palsy, (16) encephalitis and encephalopathy, (17) anxiety, (18) post‐traumatic stress disorder (PTSD), (19) psychotic disorder, (20) end‐stage renal disease (ESRD), (21) acute kidney injury and failure, (22) pancreatitis, (23) liver injury, (24) CVD death, (25) all‐cause mortality. All outcomes were diagnosed using ICD‐9‐CM for the HK cohort and ICD‐10 for the UK cohort (Table S1).

### 2.5 Baseline characteristics

The baseline characteristics included age, sex, Charlson Comorbidity Index (CCI), latest vaccination status and treatment history. All disease definitions for CCI are listed in Table S1. In the UKB dataset, ChAdOx1 and BNT162b2 COVID‐19 vaccinations were identified.^20^ In the HK‐cohort, BNT162b2 and CoronaVac COVID‐19 vaccinations were identified. Treatment history before COVID‐19 infection included chemotherapy, radiation therapy, and corticosteroid prescriptions. Treatment procedure codes and prescription codes (identified by British National Formulary chapters) are listed in Table S2.

### 2.6 Statistical analysis

To balance baseline characteristics between the exposed and unexposed groups, we applied Inverse Probability Treatment Weighting (IPTW) based on age, sex, CCI, vaccination status, chemotherapy, and radiation therapy for the UK cohort; and added corticosteroids for the HK cohort. IPTW uses propensity score by assigning weights to each individual based on the inverse probability of receiving their actual exposure (i.e., COVID‐19 infection).^20^ Patients' characteristics were described using counts and percentages for categorical variables and mean and standard deviation (SD) for continuous variables. Standardized mean difference (SMD) between exposed and unexposed groups was described, with <0.2 considered acceptable.

Incidence rates with 95% confidence interval (CI) for outcomes were calculated using Poisson distribution. Cox proportional hazard regression was conducted to evaluate the association between COVID‐19 infection and each outcome, excluding patients with a history of that particular outcome. We tested if associations were modified by age (<65 and ≥65), sex, vaccination status (fully vaccinated two doses and not fully vaccinated), CCI (<5 and ≥5), severity status and cancer duration (<5 years and ≥5 years) using interaction terms. For the UK cohort, severe cases were defined if cancer patients were admitted to intensive care unit (ICU) or hospital or used ventilatory support within 7 days of infection. For the HK cohort, severe cases were defined similarly, excluding hospital admission as all COVID‐19 cases were required to attend hospital regardless the severity of COVID‐19 infection before 2022. Cancer duration before the index date was measured in months since the diagnosis database in HK cohort only recorded diagnosis date by month before 2020. We conducted two sensitivity analyses: (1) defining the index date as 21 days post‐COVID‐19 infection; (2) conducting a competing risk analysis using the cause‐specific Cox regression to adjust for mortality, as patients might die before the occurrence of certain diseases.^21^

Two‐tailed tests were adopted, with *p* values less than 0.05 considered statistically significant. All statistical analyses were conducted using R version 4.0.3.

## 3 RESULTS

Figure 1 shows the flowchart of cancer patients' selection in HK and UK cohorts. A total of 59,299 patients with cancer was included in the UK cohort, in which 2230 (3.76%) were diagnosed with COVID‐19. A total of 68,090 patients with cancer was included in the HK cohort, in which 22,335 (32.80%) patients were diagnosed with COVID‐19. Table 1 and Table S3 summarize the baseline characteristics of the HK and UK cohort. Overall, the UK cohort was older, had a higher prevalence of multiple conditions, and a higher update rate of radiation therapy; whereas the HK cohort had a higher chemotherapy utilization rate and vaccination rate.

**TABLE 1:** Baseline characteristics of the HK and UK cohorts after weighting.

Baseline characteristics	Hong Kong			UK Biobank		
	COVID‐19 (*N* = 68,037)	Non COVID‐19 (*N* = 68,185)	SMD	COVID‐19 (*N* = 58,912)	Non COVID‐19 (*N* = 59,300)	SMD
Age, years (mean (SD))	66.6 (13.0)	66.7 (13.3)	0.001	71.5 (7.4)	71.5 (7.0)	0.001
Sex, male (%)	29,413 (43.2)	29,413 (43.1)	0.002	24,330 (41.3)	24,138 (40.7)	0.012
Charlson Comorbidity Index (mean (SD))	5.6 (2.7)	5.6 (2.7)	0.001	6.8 (2.8)	6.7 (2.8)	0.039
Myocardial infarction (%)	1006 (1.5)	803 (1.2)	0.026	4050 (6.9)	3364 (5.7)	0.050
Congestive Heart Failure (%)	1717 (2.5)	1361 (2.0)	0.035	1546 (2.6)	1262 (2.1)	0.033
Peripheral vascular disease (%)	508 (0.7)	497 (0.7)	0.002	4703 (8.0)	3320 (5.6)	0.095
Cerebrovascular disease (%)	4746 (7.0)	4033 (5.9)	0.043	7997 (13.6)	5239 (8.8)	0.151
Chronic obstructive pulmonary disease (%)	2800 (4.1)	2664 (3.9)	0.011	15,712 (26.7)	12,898 (21.8)	0.115
Dementia (%)	423 (0.6)	281 (0.4)	0.029	4443 (7.5)	1065 (1.8)	0.275
Paralysis (%)	220 (0.3)	197 (0.3)	0.006	1962 (3.3)	1187 (2.0)	0.083
Diabetes without chronic complication (%)	13,190 (19.4)	11,547 (16.9)	0.064	9466 (16.1)	7151 (12.1)	0.115
Diabetes with chronic complication (%)	1022 (1.5)	880 (1.3)	0.018	2581 (4.4)	2211 (3.7)	0.033
Chronic renal failure (%)	2156 (3.2)	1880 (2.8)	0.024	8359 (14.2)	8072 (13.6)	0.017
Mild liver disease (%)	547 (0.8)	430 (0.6)	0.021	4426 (7.5)	3553 (6.0)	0.061
Moderate–severe liver disease (%)	638 (0.9)	474 (0.7)	0.027	293 (0.5)	230 (0.4)	0.016
Ulcers (%)	2533 (3.7)	2279 (3.3)	0.021	3660 (6.2)	3405 (5.7)	0.020
Rheumatoid arthritis and other inflammatory polyarthropathies (%)	330 (0.5)	481 (0.7)	0.029	4152 (7.0)	3900 (6.6)	0.019
Acquired Immune Deficiency Syndrome (AIDS)	0 (0.00)	0 (0.00)		141 (0.2)	173 (0.3)	0.010
Malignancy (without secondary malignancy) (%)	66,940 (98.4)	67,167 (98.5)	0.010	58,553 (99.4)	58,912 (99.3)	0.006
Metastatic solid tumor (%)	10,027 (14.7)	10,717 (15.7)	0.027	6585 (11.2)	8404 (14.2)	0.090
Radiation therapy (%)	420 (0.6)	406 (0.6)	0.003	1928 (3.3)	2031 (3.4)	0.008
Chemotherapy (%)	22,302 (32.8)	22,588 (33.1)	0.007	6633 (11.3)	6544 (11.0)	0.007
Corticosteroids (%)	5708 (8.4)	5817 (8.5)	0.005			
Dose status (%)			0.005			0.013
Without vaccination record	13,407 (19.7)	13,460 (19.7)		48,870 (83.0)	49,337 (83.2)	
First dose	6435 (9.5)	6542 (9.6)		8805 (14.9)	8824 (14.9)	
Second dose	23,047 (33.9)	23,055 (33.8)		1238 (2.1)	1139 (1.9)	
Third/forth dose	25,149 (37.0)	25,128 (36.9)				

Abbreviations: SD: standard deviation; SMD: standardized mean difference.

The incidence rates and hazard ratios (HRs) for each outcome occurring 30 days post‐infection in cancer patients were summarized in Tables 2 and 3. Cancer patients with COVID‐19 infection showed higher incidence rates (per 1000 person‐years) of CVDs, acute respiratory distress syndrome and all‐cause mortality in both cohorts, as compared with those without COVID‐19 infection. COVID‐19 infection was significantly associated with higher risks of major CVDs (hazard ratio [HR] 1.8, 95% CI 1.3, 2.5 in the UK cohort; HR 1.4, 95% CI 1.1, 1.8 in the HK cohort), heart failure (HR 2.2, 95% CI 1.4, 3.3 in the UK cohort; HR 2.6, 95% CI 1.7, 4.0 in the HK cohort), CHD (HR 2.0, 95% CI 1.4, 3.0 in the UK cohort; HR 1.5, 95% CI 1.1, 2.2 in the HK cohort), CVD death (HR 4.3, 95% CI 2.9, 6.2 in the UK cohort; HR 1.7, 95% CI 1.3, 2.4 in the HK cohort), and all‐cause mortality (HR 4.7, 95% CI 4.0, 5.5 in the UK cohort; HR 1.6, 95% CI 1.5, 1.7 in the HK cohort) in both cohorts at 30‐day. Additionally, COVID‐19 infection was associated with stroke (HR 2.2, 95% CI 1.3, 3.7), myocardial infarction (HR 2.3, 95% CI 1.4, 3.8), pleurisy or pleural effusion (HR 2.0, 95% CI 1.3, 3.1), acute respiratory distress syndrome (HR 2.8, 95% CI 1.6, 4.9), interstitial lung disease (HR 3.0, 95% CI 1.6, 5.7), seizure (HR 4.3, 95% CI 1.9, 9.7), anxiety (HR 2.5, 95% CI 1.5, 3.9), ESRD (HR 2.7, 95% CI 1.2, 5.9) and acute kidney injury and failure (HR 1.9, 95% CI 1.4, 2.6) in the UK cohort.

**TABLE 2:** Incidence and hazard ratio of outcomes after the index date after weighting (UK Biobank).

	COVID‐19		Non COVID‐19		
	Event	Incidence rate^a^ (96% CI)	Event	Incidence rate^a^ (96% CI)	Hazard ratio (96% CI)
Major CVD	1137	40.30 (37.99,42.71)	790	22.91 (21.34,24.57)	**1.8 (1.3,2.5)**
Stroke	478	13.40 (12.23,14.66)	253	6.12 (5.39,6.92)	**2.2 (1.3,3.7)**
Myocardial Infarction	443	12.36 (11.24,13.57)	223	5.49 (4.79,6.26)	**2.3 (1.4,3.8)**
Heart Failure	760	21.65 (20.14,23.25)	403	9.87 (8.93,10.88)	**2.2 (1.4,3.3)**
Atrial fibrillation (and flutter)	567	17.41 (16.00,18.90)	489	12.64 (11.55,13.82)	1.4 (0.9,2.2)
CHD	807	25.61 (23.88,27.44)	471	12.85 (11.71,14.06)	**2.0 (1.4,3.0)**
Deep vein thrombosis	198	5.57 (4.82,6.41)	178	4.40 (3.78,5.09)	1.2 (0.7,2.4)
CVD Death	851	22.03 (20.57,23.56)	221	5.12 (4.47,5.84)	**4.3 (2.9,6.2)**
Pleurisy or pleural effusion	676	19.05 (17.64,20.54)	392	9.51 (8.60,10.50)	**2.0 (1.3,3.1)**
Pulmonary embolism	0	0	13	0.30 (0.16,0.52)	NA
Chronic pulmonary disease	276	9.71 (8.60,10.93)	291	8.60 (7.64,9.65)	1.1 (0.6,2.2)
Acute respiratory distress syndrome	361	9.70 (8.73,10.75)	143	3.37 (2.84,3.97)	**2.8 (1.6,4.9)**
Interstitial lung disease	322	9.21 (8.23,10.27)	128	3.18 (2.65,3.78)	**3.0 (1.6,5.7)**
Seizure	188	5.06 (4.36,5.84)	48	1.14 (0.84,1.51)	**4.3 (1.9,9.7)**
Bell's Palsy	22	0.58 (0.36,0.88)	29	0.68 (0.46,0.98)	0.8 (0.1,5.9)
Encephalitis and Encephalopathy	34	0.88 (0.61,1.23)	0	0	NA
Anxiety	693	23.75 (22.01,25.59)	332	9.38 (8.40,10.44)	**2.5 (1.5,3.9)**
PTSD	110	3.16 (2.60,3.81)	100	2.50 (2.04,3.05)	1.3 (0.5,3.5)
Psychotic disorder	67	1.76 (1.37,2.24)	34	0.79 (0.55,1.11)	2.1 (0.5,8.7)
ESRD	147	3.94 (3.33,4.64)	66	1.57 (1.21,2.00)	**2.7 (1.2,5.9)**
Acute kidney injury and failure	1254	42.46 (40.14,44.88)	798	22.26 (20.74,23.86)	**1.9 (1.4,2.6)**
Pancreatitis	61	1.59 (1.22,2.05)	35	0.82 (0.57,1.14)	2.0 (0.4,10.4)
Liver injury	0	0	4	0.09 (0.03,0.24)	NA
All‐cause mortality	4977	128.81 (125.26,132.44)	1172	27.16 (25.63,28.76)	**4.7 (4.0,5.5)**

*Note*: Major CVD: cardiovascular disease (heart failure, stroke, coronary heart disease). The bold values indicated the hazard ratio was statistically significant.

Abbreviations: CHD, coronary heart disease; CI, confidence interval; ESRD, end‐stage renal disease; NA, Not available due to insufficient number of events; PTSD, post‐traumatic stress disorder.

^a^ Incidence per 1000 persons.

**TABLE 3:** Incidence and hazard ratio of outcomes after the index date after weighting (HK cohort).

	COVID‐19		Non COVID‐19		
	Event	Incidence rate^a^ (96% CI)	Event	Incidence Rate^a^ (96% CI)	Hazard ratio (96% CI)
Major CVD	393	10.70 (9.67,11.82)	301	7.44 (6.62,8.33)	**1.4 (1.1,1.8)**
Stroke	200	5.02 (4.35,5.77)	201	4.62 (4.00,5.30)	1.1 (0.8,1.5)
Myocardial Infarction	108	2.56 (2.10,3.09)	83	1.81 (1.44,2.25)	1.4 (0.9,2.2)
Heart Failure	176	4.21 (3.61,4.88)	74	1.63 (1.28,2.05)	**2.6 (1.7,4.0)**
Atrial fibrillation (and flutter)	76	1.85 (1.46,2.31)	74	1.65 (1.30,2.08)	1.1 (0.7,1.8)
CHD	162	4.07 (3.47,4.75)	117	2.69 (2.22,3.22)	**1.5 (1.1,2.2)**
Deep vein thrombosis	37	0.87 (0.61,1.20)	33	0.72 (0.49,1.01)	1.2 (0.6,2.4)
CVD Death	230	5.37 (4.70,6.11)	140	3.02 (2.54,3.56)	**1.7 (1.3,2.4)**
Pleurisy or pleural effusion	0	0	0	0	NA
Pulmonary embolism	0	0	0	0	NA
Chronic pulmonary disease	45	1.09 (0.80,1.46)	55	1.23 (0.93,1.60)	0.9 (0.5,1.6)
Acute respiratory distress syndrome	175	4.32 (3.71,5.02)	138	3.10 (2.61,3.67)	1.4 (1.0,2.0)
Interstitial lung disease	2	0.05 (0.01,0.17)	0	0	NA
Seizure	44	1.04 (0.75,1.39)	60	1.31 (1.00,1.68)	0.8 (0.4,1.4)
Bell's Palsy	0	0	2	0.04 (0.01,0.16)	NA
Encephalitis and Encephalopathy	0	0	3	0.06 (0.01,0.19)	NA
Anxiety	3	0.07 (0.01,0.21)	3	0.07 (0.01,0.19)	1.0 (0.1,10.9)
PTSD	15	0.35 (0.20,0.58)	11	0.24 (0.12,0.43)	1.5 (0.5,4.3)
Psychotic disorder	0	0	0	0	NA
ESRD	7	0.16 (0.07,0.34)	11	0.24 (0.12,0.43)	0.7 (0.2,2.9)
Acute kidney injury and failure	65	1.54 (1.19,1.97)	71	1.55 (1.21,1.96)	1.0 (0.6,1.7)
Pancreatitis	30	0.70 (0.48,1.01)	26	0.56 (0.37,0.83)	1.3 (0.6,2.9)
Liver injury	6	0.14 (0.05,0.31)	4	0.09 (0.02,0.22)	1.6 (0.2,11.2)
All‐cause mortality	5584	130.38 (126.98,133.84)	3685	79.47 (76.93,82.08)	**1.6 (1.5,1.7)**

*Note*: Major CVD: cardiovascular disease (heart failure, stroke, coronary heart disease). The bold values indicated the hazard ratio was statistically significant.

Abbreviations: CHD, coronary heart disease; CI, confidence interval; ESRD, end‐stage renal disease; NA, not available due to insufficient number of events; PTSD, post‐traumatic stress disorder.

^a^ Incidence per 1000 persons.

Table 4 showed that age was interacted with COVID‐19 infection in risk mortality (*p* = 0.003 in UK cohort; *p* < 0.001 in HK cohort); and the vaccination status was interacted with COVID‐19 infection in risk of CVD, MI, CHD and all‐cause mortality in HK cohort. And severity status was interacted with COVID‐19 infection in risk of all‐cause mortality in both cohorts (*p* < 0.001 in UK cohort; *p* < 0.001 in HK cohort), and CVDs, anxiety, acute kidney injury and failure in HK cohort. Table S4 further showed cancer patients at an advanced age or severely infected by COVID‐19 had a higher risk of all‐cause mortality. Cancer patients who were fully vaccinated did not show significant associations of COVID‐19 with major CVD, MI and CHD; and showed a lower risk of all‐cause mortality. However, cancer patients who were severely infected were in higher risks of stroke, heart failure, CHD, deep vein thrombosis, CVD death, anxiety and acute kidney injury and failure. Sex and cancer duration did not show moderating effects on the association between COVID‐19 and morbidities; while patients with more than 5 years of cancer did not show significant associations of COVID‐19 with all‐cause mortality in HK cohort only. The sensitivity analysis that defined the index date as 21‐day after COVID‐19 infection showed similar results to the main analysis, except that acute respiratory distress syndrome became significant for the HK cohort, ESRD was not significant for the UK cohort (Table S5). The competing risk analysis also showed similar results to the main analysis (Table S6).

**TABLE 4:** *p*‐values of interaction terms.

	UK cohort						HK cohort					
	Age (<65 and ≥65)	Sex (Male and Female)	Vaccination status (<2 doses and ≥2 doses)	CCI (<5 and ≥5)	Severity	Cancer duration (<5 and ≥5 years)	Age (<65 and ≥65)	Sex (Male and Female)	Vaccination status (<2 doses and ≥2 doses)	CCI (<5 and ≥5)	Severity	Cancer duration (<5 and ≥5 years)
Major CVD	0.600	0.882	NA	0.802	0.055	0.054	0.600	0.855	**0.006**	0.478	NA	0.781
Stroke	0.539	0.514	NA	NA	**0.049**	0.797	0.886	0.420	0.678	0.412	NA	0.451
Myocardial Infarction	0.930	0.142	NA	NA	0.704	0.642	0.806	0.343	**0.010**	0.471	NA	0.579
Heart Failure	0.249	0.480	NA	0.543	**0.019**	0.978	0.258	0.359	0.866	0.978	NA	0.921
Atrial fibrillation (and flutter)	0.459	0.905	NA	NA	0.410	0.591	0.895	0.535	0.071	0.438	NA	0.903
CHD	0.654	0.255	NA	0.917	**0.040**	0.178	0.623	0.496	**0.002**	0.906	NA	0.772
Deep vein thrombosis	0.641	0.459	NA	0.810	**0.045**	0.750	0.437	0.936	0.290	0.434	NA	0.927
CVD Death	0.652	0.793	NA	NA	**0.025**	0.165	0.981	0.455	0.499	0.982	NA	0.545
Pleurisy or pleural effusion	0.199	0.855	NA	NA	0.098	0.532	NA	NA	NA	NA	NA	NA
Pulmonary embolism	NA	NA	NA	NA	NA	NA	NA	NA	NA	NA	NA	NA
Chronic pulmonary disease	0.324	0.750	NA	0.779	0.922	0.238	0.710	0.917	0.758	0.822	NA	0.296
Acute respiratory distress syndrome	0.387	0.847	NA	NA	0.507	0.931	0.081	0.068	0.335	0.058	NA	0.695
Interstitial lung disease	0.149	0.156	NA	NA	0.224	0.340	NA	NA	NA	NA	NA	NA
Seizure	0.417	0.490	NA	0.438	0.264	0.341	0.662	0.204	0.472	0.316	NA	0.202
Bell's Palsy	NA	NA	NA	NA	NA	NA	NA	NA	NA	NA	NA	NA
Encephalitis and Encephalopathy	NA	NA	NA	NA	NA	NA	NA	NA	NA	NA	NA	NA
Anxiety	0.252	0.288	NA	0.407	**0.033**	0.964	NA	NA	0.974	NA	NA	0.845
PTSD	0.469	0.326	NA	0.640	0.273	0.834	0.133	**0.014**	0.197	0.180	NA	0.343
Psychotic disorder	NA	0.833	NA	NA	NA	NA	NA	NA	NA	NA	NA	NA
ESRD	0.890	0.975	NA	NA	0.963	0.836	0.094	0.422	NA	0.092	NA	0.690
Acute kidney injury and failure	0.611	0.486	NA	0.454	**0.004**	0.168	0.818	0.154	0.560	0.975	NA	0.755
Pancreatitis	NA	NA	NA	NA	NA	NA	0.745	0.750	0.529	0.254	NA	0.801
Liver injury	NA	NA	NA	NA	NA	NA	NA	NA	NA	NA	NA	0.787
Death	**0.003**	0.666	NA	0.202	**<0.001**	0.961	**<0.001**	0.416	**0.013**	0.139	**<0.001**	**<0.001**

*Note*: Major CVD: cardiovascular disease (heart failure, stroke, coronary heart disease). The bold values indicated the hazard ratio was statistically significant.

Abbreviations: CCI, Charlson Comorbidity Index; CHD, coronary heart disease; ESRD, end‐stage renal disease; NA, not available due to insufficient number (<5 number of events); PTSD, post‐traumatic stress disorder.

## 4 DISCUSSION

The analysis of two large cohorts from UK biobank and Hong Kong demonstrated that cancer patients with COVID‐19 in both cohorts consistently had a higher risk of CVDs (particularly heart failure and CHD), CVD death and all‐cause mortality at 30 days post‐infection and beyond. The findings fill in the research gap on the post‐COVID multiorgan sequelae in cancer patients, and stress the importance of close and continuous monitoring of CVD in cancer patients following COVID‐19 infection.

While the immediate risks of COVID‐19 infection for cancer patients have been well‐documented,^22^, ^23^ our findings add to the growing body of evidence highlighting the post‐acute health consequences of COVID‐19 and the importance of closely monitoring and managing cancer patients who have had the infection. One previous study had investigated the post‐acute symptoms following COVID‐19 in cancer patients, however, it relied on clinician‐ or patient‐reported symptoms rather than formal diagnosis and included symptoms that commonly present during acute phase of COVID‐19.^24^ In contrast, our study investigated the long‐term effects of COVID‐19 on a wide range of conditions that affects various organs, with significant implications for long‐term management. Particularly, although COVID‐19 is widely recognized as a significant contributor to severe respiratory illness, acute respiratory distress syndrome was only significant as a post‐COVID sequelae occurring 21 days post‐infection in both cohorts of cancer patients in our sensitivity analysis, while it became insignificant when long COVID was defined as 30 days after infection in HK cohort.^25^ This might imply that acute respiratory distress syndrome is more of an acute symptom that typically occurs shortly after COVID‐19 infection.

Our study found, compared to omicron‐dominant HK cohort, cancer patients infected with COVID‐19 in non‐omicron‐dominant UK cohort showed higher risks of multiple post‐acute sequelae, such as stroke, myocardial infarction, pleurisy or pleural effusion, acute respiratory distress syndrome, interstitial lung disease, seizure, anxiety, ESRD and acute kidney injury and failure. The findings indicated that COVID‐19, specifically caused by a pre‐Omicron‐variant virus, might be a systematic disease associated with impairment in multiple organs.^25^ COVID‐19 contraction might also expedite the development other conditions, particularly in the UK cohort who had an older age and higher prevalence of multiple conditions.^26^, ^27^ Moreover, the risk associated with long‐term effects of COVID‐19 on CVD death and all‐cause mortality were much smaller in omicron‐dominant HK cohort as compared to non‐omicron‐dominant UK cohorts. The findings were consistent with the evidence that as the SARS‐CoV‐2 virus evolves and mutates with the emergence of newer strains such as the omicron strain, COVID‐19 progresses to milder disease characterized by a decreased risk of hospitalization and mortality.^28^ The higher risk of post‐infection sequelae in the UK cohort might also be explained by its low vaccination rate, which was confirmed by our subgroup analysis showing that full vaccination moderated the association between COVID‐19 and outcomes. Moreover, COVID‐19 exerted higher risk of mortality than most morbidities in the UK cohort of cancer patients, except seizure, acute respiratory distress syndrome, interstitial lung disease and ESRD; whereas such findings were not observed in the HK cohort. This might be because the UK cohort had a much shorter follow up period, and patients with milder symptoms of morbidities might not have sought medical advice yet, which caused underestimation of morbidities risk after acute phase of COVID‐19. However, cancer patients in both cohorts were found to incur a similar risk of CVD risk, suggesting that the risk of long COVID is still significant even with milder strain of the virus. Given that the Omicron variant affects most people particularly in Hong Kong due to its high transmissibility, the long‐term consequences of COVID‐19 continue to pose a significant threat to public health.^10^

Our findings of increased CVD risk in the post‐acute phase of COVID‐19 among cancer patients are consistent with previously reported evidence in the general population. For instance, a study conducted by Al‐Aly et al. demonstrated a higher risk of cardiovascular complications, such as heart failure, myocardial infarction, and stroke, among COVID‐19 survivors within 6 months of infection compared to the control group.^4^ Another study by Puntmann et al. reported that 78% of COVID‐19 patients showed ongoing cardiovascular involvement, and 60% had ongoing myocardial inflammation, as assessed by cardiac magnetic resonance imaging, two to three months after the initial diagnosis.^5^ The exact mechanisms underlying the increased CVD risk in the post‐acute phase of COVID‐19 among cancer patients are not yet fully understood. Potential factors include the direct impact of the virus on the cardiovascular system, systemic inflammation, or exacerbation of pre‐existing conditions.^29^, ^30^ Additionally, cancer patients may be more susceptible to CVD due to their underlying malignancy, cancer treatments, or weakened immune systems.

An increased risk of mental/psychiatric conditions (i.e. anxiety and seizure) in UK cohort was identified. Our study was among the first to demonstrate the link between COVID‐19 and psychiatric conditions at around one‐month post‐infection among cancer patients. The findings were consistent with previous study using electronic health records of primarily non‐cancer patients in the United States, which showed those with COVID‐19 had a higher risk of developing neurological and psychiatric diseases 6 months following the infection.^31^ However, such relationship was insignificant in HK cohort. There were two possible explanations. First, as the UK cohort was primarily affected by pre‐Omicron variants, we might reasonably assume that these patients infected with COVID‐19 had more severe symptoms than COVID‐19 cases in Hong Kong who were mainly caused by the Omicron variant. And severe infection was the reason for neurological and psychiatric morbidities, which was supported by previous studies and our findings of the moderating effects of initial infection severity.^31^ Second, the HK cohort had a low incidence rate of mental/psychiatric conditions, resulting in a reduced statistical power. The findings broaden our understanding of the potential post‐acute mental health consequences of the virus, and highlight the potential influence of different viral strains on post‐acute outcomes among cancer patient.

The risk of post‐COVID sequelae varies widely based on factors such as age, vaccination status, comorbidities, and initial infection severity, as shown in the subgroup analyses. Older and incompletely vaccinated cancer patients experienced higher risks of all‐cause mortality, major CVD, MI, and CHD. These findings emphasize the need for tailored preventative measures and treatments, considering age and cancer status. Full vaccination possibly plays a vital role in reducing COVID‐19‐related adverse cardiovascular effects in cancer patients. In addition, our study found patients with more than 5 years of cancer did not show significant associations of COVID‐19 with all‐cause mortality, whereas patients with less than 5 years of cancer did in HK cohort; while the UK cohort did not find moderating effects of cancer duration. More research is warranted to explain the results since the severity of cancer and how cancer treatment is administrated during the entire cancer experience is unclear. Healthcare providers should monitor cancer patients for long COVID symptoms, providing appropriate support and care. The long‐term effects of COVID‐19 in cancer patients remain an area of active research and concern. Continued study is crucial for optimizing care and outcomes even after the pandemic, as the virus will likely persist, causing sporadic infections and outbreaks worldwide.

## 5 LIMITATIONS

Several limitations should be noted. First is the inconsistent definition of severe COVID‐19 in the HK and UK cohorts. Since all COVID‐19 cases were admitted to hospital before 2022 in HK, we only defined severe COVID‐19 by ICU admission and the use of ventilatory support in the HK cohort. Hence only a few severe cases were identified, resulting a lack of statistical power. Second, the UK cohort recruited participants in the early phase of COVID‐19 epidemic, resulting in a small number of fully vaccinated individuals. Third is the lack of long‐term follow‐up data. Future studies with longer follow‐up periods are needed to fully understand the long‐term impact of COVID‐19 on cancer patients. Additionally, our study was limited to two regions, the United Kingdom and Hong Kong, and may not be generalizable to other populations. Further research is needed to confirm our findings in other populations.

## 6 CONCLUSIONS

In conclusion, our study highlights the increased risk of post‐acute phase complications, particularly CVDs, among cancer patients with COVID‐19. Healthcare providers should be vigilant in monitoring these patients for potential long‐term health complications and prioritize preventive measures to mitigate the risk of post‐acute sequelae. These findings have important implications for the long‐term management of cancer patients during the COVID‐19 pandemic and beyond and could help in service planning and identification of research priorities.

## AUTHOR CONTRIBUTIONS

**Carlos King Ho Wong:** Validation (equal); writing – review and editing (equal). **Celine Sze Ling Chui:** Validation (equal); writing – review and editing (equal). **Esther Wai Yin Chan:** Validation (equal); writing – review and editing (equal). **Francisco Tsz Tsun Lai:** Validation (equal); writing – review and editing (equal). **Jiayi Zhou:** Formal analysis (equal); validation (equal); writing – original draft (equal); writing – review and editing (equal). **Shing Fung Lee:** Validation (equal); writing – original draft (equal); writing – review and editing (equal). **Xue Li:** Validation (equal); writing – review and editing (equal). **Vincent Ka Chun Yan:** Formal analysis (equal); validation (equal); writing – review and editing (equal). **Ian Chi Kei Wong:** Conceptualization (equal); funding acquisition (lead); project administration (equal); supervision (equal); validation (equal); writing – review and editing (equal). **Eric Yuk Fai Wan:** Conceptualization (equal); data curation (lead); formal analysis (equal); methodology (equal); project administration (equal); supervision (equal); validation (equal); writing – original draft (equal); writing – review and editing (equal).

## FUNDING INFORMATION

The Health Bureau and University Grants Committee's Collaborative Research Fund, both under the Government of the Hong Kong Special Administrative Region, supported the study. The grant numbers are COVID19F01 and C7154‐20GF. Despite their financial support, the funders had no involvement in the study's design, execution, data collection and analysis, results interpretation, or the manuscript's creation, review and submission process. As D24H partly funded FTTL and ICKW's positions, AIR@InnoHK managed by Innovation and Technology Commission, partly supported this study.

## CONFLICT OF INTEREST STATEMENT

EYFW reports research fundings from the Health Bureau of the Hong Kong Special Administrative Region, and the Hong Kong Research Grants Council, which were unrelated to this study. FTTL reports support from the RGC Postdoctoral Fellowship under the Hong Kong Research Grants Council and research grants from the Health Bureau of the Hong Kong Special Administrative Region, which were unrelated to this study. CSLC reports grants from the Health Bureau of the Hong Kong Government, Hong Kong Research Grant Council, Hong Kong Innovation and Technology Commission, Pfizer, IQVIA, and Amgen, as well as personal fees from PrimeVigilance; all unrelated to this study. XL reports grants from the Health Bureau of the Hong Kong Special Administrative Region, Janssen and Pfizer, as well as internal funding from the University of Hong Kong, and consultancy fees from Merck Sharp &amp, Dohme, all outside this study. EWC reports grants from Research Grants Council (RGC, Hong Kong), Research Fund Secretariat of the Health Bureau, National Natural Science Fund of China, Wellcome Trust, Bayer, Bristol‐Myers Squibb, Pfizer, Janssen, Amgen, Takeda, and Narcotics Division of the Security Bureau of the Hong Kong Special Administrative Region; honorarium from Hospital Authority; outside the submitted work. ICKW reports funding from Amgen, Bristol Myers Squibb, Pfizer, Janssen, Bayer, GSK, Novartis, the Hong Kong Research Grants Council, the Hong Kong Health and Medical Research Fund, the National Institute for Health Research in England, the European Commission, and the National Health and Medical Research Council in Australia, outside the submitted work. He is a non‐executive director of Jacobson Medical in Hong Kong and a consultant to IQVIA and World Health Organization. The other authors declare that they have no competing interests.

## ETHICS STATEMENT

This study was approved by the Institutional Review Board of the University of Hong Kong/Hospital Authority Hong Kong West Cluster (UW20‐556 and UW21‐149) and Department of Health, HK (L/M21/2021). Informed consent was exempted as data were pseudo‐anonymized and patients' confidentiality was maintained. The North West Multi‐Centre Research Ethics Committee issued separate ethical approval for the data extraction from UKB (Application No. 65688). Written consent was obtained from all participants in UKB at the time of recruitment, and those who withdrew from the study were excluded from the analysis.

## CONSENT

The competent authorities exempted informed consent from participants as patient data was anonymized.

## Supporting information


Table S1.

## Data Availability

As the data includes confidential information and is subject to third‐party use restrictions, it cannot be shared with the public.
