# Supplementary material for: Post‐acute sequelae of COVID‐19 in cancer patients: Two cohorts in UK and Hong Kong
Source: Cancer Med. 2024 Dec 7;13(23):e70134. doi: 10.1002/cam4.70134 (PMC11624603; doi:10.1002/cam4.70134)
Supplement: Supplementary file 1 — Table S1. [file CAM4-13-e70134-s001.docx]

**Supplementary Materials**

**Table S1.** Disease definition

| **Disease** | **ICD-9-CM** | **ICD-10** |
| --- | --- | --- |
| Myocardial infarction | 410 | I21.x, I22.x, I23.x, I24.1, I25.2 |
| Heart failure | 428,398.91,402.01,402.11,402.91,404.01,404.03, 404.11,404.13,404.91,404.93 | I50.x |
| Stroke | 430-438 | I60.x, I61.x, I63.x, I64.x |
| Atrial fibrillation | 427.3 | I48.x |
| coronary heart disease | 410-414, 36.0, 36.1 | I20.x - I25.x, I46.x |
| Deep vein thrombosis | 453 | I80-I81, I82.Ax, I82.Bx, I82.Cx |
| pleurisy or pleural effusion | 511.9 | J90,J91 |
| Pulmonary embolism | 415.3 | I26 |
| Chronic pulmonary disease | 490-496 | J40.x-J47.x, J60.x-J67.x, J68.4, J70.1, J70.3 |
| Acute respiratory distress syndrome | 518.8x, 518.51-518.53 | J96.0, J96.10, J96.2, J80, R06.03, J98.4, J95.1, J95.2, J95.3, J95.821, J95.822 |
| Interstitial lung disease | 515, 516.3, 517.2, 517.8, 714.81, 135, 495 | M05.1, J84, J81, J82 |
| Seizure | 333.2, 345, 649.4, 780.3, 779.0, 780.3 | G40-G41 |
| Bell’s Palsy | 351.1, 351.8, 351.9 | G51.1,G51.2,G51.4,G51.8,G51.9 |
| Encephalitis and Encephalopathy | 323.0-323.9, 348.30, 348.31, 348.39 | G05.3, G04.9, G04.91, G93.41, I67.83 |
| Anxiety | 300.0 | F40-F41 |
| Post-traumatic stress disorder | 308.x-309.x | F43.0, R45.7, F43.20, F43.21, F432.4, F43.25 |
| Psychotic disorder | 298.8, 298.9 | F23, F29 |
| End stage renal disease | 585.6, 586 | I12.0. I13.x, N18.5, N18.6, Z99.2, N19.x, Z94.0, Z49.0 |
| Acute kidney injury | 584.5-584.9 | N17-N19 |
| Pancreatitis | 577.0, 577.1 | K85.0x, K85.8x, K85.9x |
| Liver injury | 570, 573.3 | K72.00, K76.2, K71.6, K75.9 |
| Congestive heart failure | 398.91, 402.01, 402.11, 402.91, 404.01, 404.03, 404.11, 404.13, 404.91, 404.93, 428 | I11.0, I13.0 I13.2, I25.5,  I42.0, I42.5-I43.x, I50.x, P29.0 |
| Peripheral vascular disease | 441, 443.9, 785.4 | I70.x, I71.x, I73.1, I73.8, I73.9,  I77.1, I79.0, I79.1, I79.8, K55.1,  K55.8, K55.9, Z95.8, Z95.9 |
| Cerebrovascular disease | 430-438 | G45.x, G46.x, H34.0x, H34.1x, H34.2x, I60.x-I68.x |
| Chronic obstructive pulmonary disease (%) | 490-496, 500-505, 506.4 | J40.x-J47.x, J60.x-J67.x, J68.4, J70.1, J70.3 |
| Dementia | 290 | F01.x-F03.x, F04, F05, F06.1, F06.8, G13.2, G13.8, G30.x,G31.1,G31.2,G91.4,G94,R41.81,R54 |
| Paralysis | 342, 344.1 | G04.1, G11.4, G80.0, G80.1, G80.2, G81.x,G82.x, G83.x |
| Diabetes without chronic complications | 250.0-250.3, 250.7 | E08.0x, E08.1x, E08.6x, E08.8x, E08.9x, E09.0x, E09.1x, E09.6x, E09.8x, E09.9x, E10.0x, E10.1x, E10.6x, E10.8x, E10.9x, E11.0x, E11.1x, E11.6x, E11.8x, E11.9x, E13.0x, E13.1x, E13.6x, E13.8x, E13.9x, |
| Diabetes with chronic complications | 250.4-250.6 | E08.2, E08.3, E08.4, E08.5, E09.2, E09.3, E09.4, E09.5, E10.2, E10.3, E10.4, E10.5, E11.2, E11.3, E11.4, E11.5, E13.2, E13.3, E13.4, E13.5 |
| Chronic renal failure | 582, 585, 586, 588, 583.0-583.2, 583.4, 583.6, 583.7 | N03.x, N18.1- N18.9, N19, N25, N05.9, N05.2, N05.5, N17.1, N17.2 |
| Liver disease, mild | 571.2, 571.4-571.6 | B18.x, K70.0-K70.3, K70.9, K71.3, K71.4, K71.5, K71.7, K73.x, k74.x, K76.0, K76.2, K76.3, K76.4, K76.8, K76.9, Z94.4 |
| Liver disease, moderate to severe | 456.0-456.2, 572.2-572.4. 572.8 | I85.0x, I86.4, K70.4x, K71.1x, K72.1x, K72.9x, K76.5, K76.6, K76.7 |
| Ulcers | 531-534 | k25.x-k28.x |
| Rheumatoid arthritis and other inflammatory polyarthropathies | 710.0, 710.1, 710.4, 714.0, 714.1, 714.2, 714.81, 725 | M05.x, M06.x, M31.5, M32.x-M34.x, M35.1, M35.3, M36.0 |
| Acquired Immune Deficiency Syndrome | 042 | B20.x, R64.0, G93.4 |
| Cancer | 140-171, 174--209 | C00.x – C42.x, C45.x-C96.x |
| Malignancy (without secondary malignancy) | 140-149, 150-159, 180-189, 170-172, 174, 175, 176, 179, 160-165, 190-195, 200-208 | C00.x – C42.x, C45.x-C76.x, C80.x-C96.x |
| Metastatic solid tumour | 196-199 | C77.x - C79.x, C80.0, C80.2 |

Note: ICD-9-CM: International Classification of Diseases, Ninth Revision, clinical modification, ICD-10: International Classification of Diseases, Tenth Revision

**Table S2.** Treatment definitions

| **Treatment** | **ICD-9-CM Procedure Code** | **ICD10** | **BNF Chapter** |
| --- | --- | --- | --- |
| Chemotherapy | 99.25 | Z51.1, Z51.11, Z51.12 | 8.1, 8.2 |
| Radiation therapy | 92.2-92.39 | Z51.0 |  |
| Corticosteroids |  |  | 6.3.1, 6.3.2 |

Note: ICD-9-CM: International Classification of Diseases, Ninth Revision, clinical modification, ICD-10: International Classification of Diseases, Tenth Revision, BNF: British National Formulary

**Table S3.** Baseline characteristics of the HK and UK cohorts before weighting

|  | Hong Kong | | | UK Biobank | | |
| --- | --- | --- | --- | --- | --- | --- |
| **Baseline characteristics** | **COVID-19 (N=22,335)** | **Non COVID-19 (N=45,755)** | **SMD** | **COVID-19 (N=2,230)** | **Non COVID-19 (N=57,069)** | **SMD** |
| Age, years (mean (SD)) | 67.1 (13.1) | 66.5 (13.3) | 0.039 | 69.6 (8.0) | 71.6 (6.9) | 0.306 |
| Sex, male (%) | 10894 (48.8) | 18393 (40.2) | 0.173 | 968 (43.4) | 23169 (40.6) | 0.030 |
| Charlson Comorbidity Index (mean (SD)) | 5.9 (2.8) | 5.5 (2.6) | 0.149 | 7.3 (3.3) | 6.7 (2.7) | 0.088 |
| Myocardial infarction (%) | 385 (1.7) | 499 (1.1) | 0.054 | 181 (8.1) | 3215 (5.6) | 0.116 |
| Congestive Heart Failure (%) | 657 (2.9) | 850 (1.9) | 0.071 | 63 (2.8) | 1204 (2.1) | 0.056 |
| Peripheral vascular disease (%) | 192 (0.9) | 314 (0.7) | 0.020 | 206 (9.2) | 3170 (5.6) | 0.070 |
| Cerebrovascular disease (%) | 1706 (7.6) | 2567 (5.6) | 0.082 | 323 (14.5) | 5022 (8.8) | 0.163 |
| Chronic obstructive pulmonary disease (%) | 1089 (4.9) | 1611 (3.5) | 0.068 | 640 (28.7) | 12357 (21.7) | 0.120 |
| Dementia (%) | 161 (0.7) | 182 (0.4) | 0.043 | 157 (7.0) | 1025 (1.8) | 0.238 |
| Paralysis (%) | 87 (0.4) | 121 (0.3) | 0.022 | 98 (4.4) | 1125 (2.0) | 0.163 |
| Diabetes without chronic complication (%) | 4441 (19.9) | 7697 (16.8) | 0.079 | 414 (18.6) | 6835 (12.0) | 0.074 |
| Diabetes with chronic complication (%) | 368 (1.6) | 564 (1.2) | 0.035 | 126 (5.7) | 2109 (3.7) | 0.001 |
| Chronic renal failure (%) | 810 (3.6) | 1142 (2.5) | 0.066 | 368 (16.5) | 7720 (13.5) | 0.050 |
| Mild liver disease (%) | 195 (0.9) | 274 (0.6) | 0.032 | 215 (9.6) | 3381 (5.9) | 0.110 |
| Moderate-severe liver disease (%) | 230 (1.0) | 287 (0.6) | 0.044 | 18 (0.8) | 214 (0.4) | 0.073 |
| Ulcers (%) | 927 (4.2) | 1442 (3.2) | 0.053 | 155 (7.0) | 3257 (5.7) | 0.046 |
| Rheumatoid arthritis and other inflammatory polyarthropathies (%) | 140 (0.6) | 253 (0.6) | 0.010 | 165 (7.4) | 3746 (6.6) | 0.043 |
| Acquired Immune Deficiency Syndrome (AIDS) | 0 (0.0) | 0 (0.0) | . | 13 (0.6) | 158 (0.3) | 0.033 |
| Malignancy (without secondary malignancy) (%) | 21944 (98.2) | 45094 (98.6) | 0.024 | 2212 (99.2) | 56701 (99.4) | 0.006 |
| Metastatic solid tumour (%) | 3963 (17.7) | 6380 (13.9) | 0.104 | 410 (18.4) | 7882 (13.8) | 0.053 |
| Radiation therapy (%) | 109 (0.5) | 291 (0.6) | 0.020 | 77 (3.5) | 1954 (3.4) | 0.170 |
| Chemotherapy (%) | 8500 (38.1) | 13960 (30.5) | 0.159 | 253 (11.3) | 6291 (11.0) | 0.096 |
| Corticosteroids (%) | 3714 (16.6) | 1973 (4.3) | 0.411 |  |  |  |
| Dose status (%) |  |  | 0.348 |  |  | 0.205 |
| Without vaccination record | 4877 (21.8) | 8798 (19.2) |  | 2004 (89.9) | 47332 (82.9) |  |
| First dose | 3330 (14.9) | 3080 (6.7) |  | 194 (8.7) | 8630 (15.1) |  |
| Second dose | 7888 (35.3) | 15028 (32.8) |  | 32 (1.4) | 1107 (1.9) |  |
| Third/ forth dose | 6240 (27.9) | 18849 (41.2) |  |  |  |  |
| Cancer duration, months (mean (SD)) | 33.3 (23.2) | 51.9 (15.1) | 0.952 | 122.9 (109.6) | 139.5(108.8) | 0.153 |

Note: SMD: standardized mean difference; SD: standard deviation.

**Table S4.** Hazard ratio for outcomes in different groups of cancer patients

|  | **UK cohort** | | **HK cohort** | |
| --- | --- | --- | --- | --- |
|  | Hazard Ratio (95% CI) | P for interaction term | Hazard Ratio (95% CI) | P for interaction term |
| All-cause mortality, age <65 | 2.1 (1.2,3.6) | 0.003 | 1.3 (1.1,1.4) | <0.001 |
| All-cause mortality, age ≥65 | 5.1 (4.3,6.0) |  | 1.8 (1.7,1.9) |  |
| Major CVD, vaccination dose <2 | 1.8 (1.3,2.6) | NA | 2.2 (1.5,3.2) | 0.006 |
| Major CVD, vaccination dose ≥2 | NA |  | 1.1 (0.8,1.5) |  |
| MI, vaccination dose <2 | 2.3 (1.4,3.8) | NA | 2.9 (1.4,5.7) | 0.010 |
| MI, vaccination dose ≥2 | NA |  | 0.9 (0.5,1.6) |  |
| CHD, vaccination dose <2 | 2.0 (1.4,3.0) | NA | 3.3 (1.8,6.1) | 0.002 |
| CHD, vaccination dose ≥2 | NA |  | 1.0 (0.6,1.6) |  |
| All-cause mortality, vaccination dose <2 | 4.7 (4.0,5.5) | NA | 1.7 (1.6,1.9) | 0.013 |
| All-cause mortality, vaccination dose ≥2 | NA |  | 1.5 (1.3,1.6) |  |
| Major CVD, non-severe | 1.5 (0.9,2.3) | 0.055 | 1.5 (1.2,1.9) | NA |
| Major CVD, severe | 3.0 (1.7,5.3) |  | NA |  |
| Stroke, non-severe | 1.6 (0.8,3.3) | 0.049 | 1.1 (0.8,1.5) | NA |
| Stroke, severe | 4.4 (2.1,9.1) |  | NA |  |
| Heart failure, non-severe | 1.6 (0.9,2.9) | 0.019 | 2.6 (1.7,3.9) | NA |
| Heart failure, severe | 4.3 (2.4,7.9) |  | NA |  |
| CHD, non-severe | 1.6 (0.9,2.6) | 0.040 | 1.6 (1.1,2.3) | NA |
| CHD, severe | 3.6 (2.0,6.6) |  | NA |  |
| Deep vein thrombosis, non-severe | 0.8 (0.3,2.1) | 0.045 | 1.2 (0.6,2.4) | NA |
| Deep vein thrombosis, severe | 3.0 (1.2,7.1) |  | NA |  |
| CVD Death, non-severe | 3.2 (1.9,5.3) | 0.025 | 1.8 (1.3,2.4) | NA |
| CVD Death, severe | 7.4 (4.3,12.8) |  | NA |  |
| Anxiety, non-severe | 1.8 (0.9,3.5) | 0.033 | NA | NA |
| Anxiety, severe | 5.0 (2.6,9.3) |  | NA |  |
| Acute kidney injury and failure, non-severe | 1.4 (0.9,2.2) | 0.004 | 1.0 (0.6,1.7) | NA |
| Acute kidney injury and failure, severe | 3.8 (2.3,6.1) |  | NA |  |
| All-cause mortality, non-severe | 3.4 (2.8,4.3) | <0.001 | 1.2 (1.1,1.3) | <0.001 |
| All-cause mortality, severe | 8.5 (6.7,10.8) |  | 4.1 (3.7,4.7) |  |
| All-cause mortality, cancer <5 years | 4.4 (3.5,5.4) | 0.961 | 1.7 (1.5,1.8) | <0.001 |
| All-cause mortality, cancer ≥5 years | 4.4 (3.5,5.6) |  | 1.0 (0.8,1.2) |  |

Note: Major CVD: cardiovascular disease (heart failure, stroke, coronary heart disease); MI: Myocardial Infarction; CHD: coronary heart disease; CI: confidence interval; NA: Not available due to insufficient number (<5 number of events)

**Table S5a.** Incidence rate and hazard ratio of outcomes after the index date that was redefined as 21-day after COVID-19 infection after weighting (UK Biobank)

|  | **COVID-19** | | **Control** | | |
| --- | --- | --- | --- | --- | --- |
|  | Event | Incidence Rate† (96% CI) | Event | Incidence Rate† (96% CI) | Hazard Ratio (96% CI) |
| Major CVD | 1235 | 42.90 (40.54,45.36) | 805 | 22.56 (21.03,24.17) | **1.9 (1.4,2.7)** |
| Stroke | 507 | 13.96 (12.77,15.23) | 255 | 5.96 (5.25,6.74) | **2.3 (1.4,3.9)** |
| Myocardial Infarction | 436 | 11.95 (10.85,13.13) | 226 | 5.38 (4.70,6.13) | **2.3 (1.3,3.8)** |
| Heart Failure | 894 | 25.06 (23.44,26.76) | 417 | 9.88 (8.95,10.87) | **2.5 (1.7,3.7)** |
| Atrial fibrillation (and flutter) | 573 | 17.29 (15.90,18.76) | 501 | 12.53 (11.45,13.67) | 1.4 (0.9,2.2) |
| CHD | 846 | 26.32 (24.58,28.16) | 479 | 12.63 (11.53,13.82) | **2.1 (1.4,3.1)** |
| Deep vein thrombosis | 230 | 6.36 (5.57,7.24) | 174 | 4.16 (3.56,4.82) | 1.5 (0.8,2.8) |
| CVD Death | 1018 | 25.91 (24.34,27.55) | 233 | 5.22 (4.57,5.93) | **4.9 (3.5,6.9)** |
| Pleurisy or pleural effusion | 650 | 18.01 (16.65,19.45) | 405 | 9.50 (8.60,10.47) | **1.9 (1.2,2.9)** |
| Pulmonary embolism | 0 | 0 | 15 | 0.34 (0.19,0.55) | NA |
| chronic pulmonary disease | 303 | 10.48 (9.33,11.72) | 294 | 8.40 (7.47,9.42) | 1.3 (0.7,2.3) |
| Acute respiratory distress syndrome | 375 | 9.91 (8.93,10.96) | 145 | 3.31 (2.79,3.89) | **2.9 (1.7,5.2)** |
| Interstitial lung disease | 426 | 11.98 (10.87,13.17) | 129 | 3.10 (2.59,3.68) | **3.9 (2.3,6.8)** |
| Seizure | 173 | 4.58 (3.92,5.32) | 51 | 1.17 (0.87,1.54) | **3.8 (1.7,8.7)** |
| Bell's Palsy | 21 | 0.54 (0.34,0.83) | 30 | 0.68 (0.46,0.97) | 0.8 (0.1,5.6) |
| Encephalitis and Encephalopathy | 33 | 0.84 (0.58,1.18) | 0 | 0 | NA |
| Anxiety | 672 | 22.55 (20.88,24.32) | 346 | 9.45 (8.48,10.50) | **2.3 (1.5,3.7)** |
| PTSD | 111 | 3.14 (2.58,3.78) | 100 | 2.42 (1.97,2.94) | 1.4 (0.5,3.6) |
| Psychotic disorder | 65 | 1.68 (1.30,2.14) | 36 | 0.81 (0.57,1.12) | 2.0 (0.5,8.2) |
| ESRD | 120 | 3.17 (2.62,3.78) | 72 | 1.66 (1.30,2.09) | 1.9 (0.9,4.4) |
| Acute kidney injury and failure | 1300 | 43.10 (40.79,45.51) | 826 | 22.27 (20.78,23.84) | **1.9 (1.4,2.6)** |
| Pancreatitis | 60 | 1.54 (1.18,1.98) | 36 | 0.81 (0.57,1.12) | 1.9 (0.4,10.1) |
| Liver injury | 0 | 0 | 5 | 0.11 (0.04,0.26) | NA |
| All-cause mortality | 5859 | 149.11 (145.31,152.97) | 1207 | 27.04 (25.53,28.61) | **5.4 (4.6,6.2)** |

Note: Major CVD: cardiovascular disease (heart failure, stroke, coronary heart disease); CHD: coronary heart disease; PTSD: post-traumatic stress disorder; ESRD: end-stage renal disease; CI: confidence interval; NA: Not available due to insufficient number of events

**Table S5b.** Incidence rate and hazard ratio of outcomes after the index date that was redefined as 21-day after COVID-19 infection after weighting (HK cohort)

|  | **COVID-19** | | **Control** | | |
| --- | --- | --- | --- | --- | --- |
|  | Event | Incidence Rate† (96% CI) | Event | Incidence Rate† (96% CI) | Hazard Ratio (96% CI) |
| Major CVD | 424 | 11.09 (10.06,12.20) | 312 | 7.43 (6.63,8.30) | **1.5 (1.2,1.9)** |
| Stroke | 209 | 5.04 (4.38,5.77) | 206 | 4.56 (3.96,5.23) | 1.1 (0.8,1.5) |
| Myocardial Infarction | 112 | 2.54 (2.10,3.06) | 88 | 1.85 (1.49,2.28) | 1.4 (0.9,2.1) |
| Heart Failure | 195 | 4.48 (3.87,5.15) | 77 | 1.63 (1.29,2.04) | **2.8 (1.9,4.1)** |
| Atrial fibrillation (and flutter) | 90 | 2.10 (1.69,2.58) | 75 | 1.62 (1.27,2.02) | 1.3 (0.8,2.1) |
| CHD | 173 | 4.17 (3.57,4.84) | 124 | 2.75 (2.29,3.28) | **1.5 (1.1,2.1)** |
| Deep vein thrombosis | 40 | 0.90 (0.65,1.23) | 40 | 0.84 (0.60,1.14) | 1.1 (0.5,2.1) |
| CVD Death | 248 | 5.56 (4.89,6.29) | 154 | 3.20 (2.72,3.75) | **1.7 (1.3,2.3)** |
| Pleurisy or pleural effusion | 0 | 0 | 0 | 0 | NA |
| Pulmonary embolism | 0 | 0 | 0 | 0 | NA |
| Chronic pulmonary disease | 45 | 1.05 (0.76,1.40) | 52 | 1.12 (0.84,1.47) | 0.9 (0.5,1.7) |
| Acute respiratory distress syndrome | 189 | 4.48 (3.87,5.17) | 142 | 3.08 (2.59,3.63) | **1.5 (1.0,2.0)** |
| Interstitial lung disease | 2 | 0.04 (0.01,0.16) | 0 | 0 | NA |
| Seizure | 51 | 1.15 (0.86,1.52) | 63 | 1.32 (1.02,1.69) | 0.9 (0.5,1.5) |
| Bell's Palsy | 0 | 0 | 2 | 0.04 (0.01,0.15) | NA |
| Encephalitis and Encephalopathy | 0 | 0 | 3 | 0.06 (0.01,0.18) | NA |
| Anxiety | 3 | 0.07 (0.01,0.20) | 3 | 0.06 (0.01,0.18) | 0.9 (0.1,10.8) |
| PTSD | 19 | 0.43 (0.26,0.67) | 11 | 0.23 (0.12,0.41) | 1.8 (0.7,5.0) |
| Psychotic disorder | 0 | 0 | 0 | 0 | NA |
| ESRD | 10 | 0.22 (0.11,0.41) | 15 | 0.31 (0.17,0.52) | 0.7 (0.2,2.4) |
| Acute kidney injury and failure | 70 | 1.59 (1.24,2.01) | 75 | 1.58 (1.24,1.98) | 1.0 (0.6,1.7) |
| Pancreatitis | 31 | 0.70 (0.47,0.99) | 27 | 0.56 (0.37,0.82) | 1.2 (0.5,2.7) |
| Liver injury | 6 | 0.13 (0.05,0.29) | 7 | 0.15 (0.06,0.30) | 0.9 (0.1,5.4) |
| All-cause mortality | 6172 | 138.27 (134.84,141.76) | 3802 | 79.06 (76.57,81.62) | **1.7 (1.6,1.8)** |

Note: Major CVD: cardiovascular disease (heart failure, stroke, coronary heart disease); CHD: coronary heart disease; PTSD: post-traumatic stress disorder; ESRD: end-stage renal disease; CI: confidence interval; NA: Not available due to insufficient number of events

**Table S6.** Competing risk analysis

|  | UK cohort | HK cohort |
| --- | --- | --- |
|  | Hazard Ratio (95% CI) | Hazard Ratio (95% CI) |
| Major CVD | **1.7 (1.2,2.5)** | **1.4 (1.1,1.8)** |
| Stroke | **2.1 (1.2,3.5)** | 1.1 (0.8,1.4) |
| Myocardial Infarction | **2.2 (1.3,3.7)** | 1.4 (0.9,2.1) |
| Heart Failure | **2.1 (1.4,3.2)** | **2.5 (1.7,3.9)** |
| Atrial fibrillation (and flutter) | 1.3 (0.8,2.1) | 1.1 (0.7,1.8) |
| CHD | **1.9 (1.3,2.9)** | **1.5 (1.0,2.1)** |
| Deep vein thrombosis | 1.2 (0.6,2.3) | 1.1 (0.6,2.3) |
| CVD Death | **4.1 (2.8,5.9)** | **1.7 (1.2,2.3)** |
| Pleurisy or pleural effusion | **1.9 (1.3,2.9)** | NA |
| Pulmonary embolism | NA | NA |
| chronic pulmonary disease | 1.1 (0.6,2.1) | 0.9 (0.5,1.6) |
| Acute respiratory distress syndrome | **2.7 (1.5,4.7)** | 1.4 (1.0,1.9) |
| Interstitial lung disease | **2.9 (1.5,5.3)** | NA |
| Seizure | **4.1 (1.8,9.2)** | 0.8 (0.4,1.4) |
| Bell's Palsy | 0.8 (0.1,5.6) | NA |
| Encephalitis and Encephalopathy | NA | NA |
| Anxiety | **2.3 (1.5,3.7)** | 0.9 (0.1,10.6) |
| PTSD | 1.2 (0.5,3.2) | 1.5 (0.5,4.2) |
| Psychotic disorder | 2.0 (0.5,8.3) | NA |
| ESR | **2.5 (1.1,5.5)** | 0.7 (0.2,2.8) |
| Acute kidney injury and failure | **1.8 (1.3,2.5)** | 1.0 (0.6,1.6) |
| Pancreatitis | 1.9 (0.4,9.7) | 1.2 (0.5,2.9) |
| Liver injury | NA | 1.6 (0.2,11.1) |

Note: Major CVD: cardiovascular disease (heart failure, stroke, coronary heart disease); CHD: coronary heart disease; PTSD: post-traumatic stress disorder; ESRD: end-stage renal disease; CI: confidence interval; NA: Not available due to insufficient number of events
